# Genome Sequence of *Staphylococcus saprophyticus* DPC5671, a Strain Isolated from Cheddar Cheese

**DOI:** 10.1128/genomeA.00193-17

**Published:** 2017-04-20

**Authors:** Andrea S. Bertuzzi, Caitriona M. Guinane, Fiona Crispie, Kieran N. Kilcawley, Paul L. H. McSweeney, Mary C. Rea

**Affiliations:** a Teagasc Food Research Centre, Moorepark, Fermoy, Cork, Ireland; b School of Food and Nutritional Science, University College Cork, Cork, Ireland; c Department of Biological Sciences, Cork Institute of Technology, Cork, Ireland; d APC Microbiome Institute, University College Cork, Cork, Ireland

## Abstract

The draft genome sequence of *Staphylococcus saprophyticus* DPC5671, isolated from cheddar cheese, was determined. *S. saprophyticus* is a common Gram-positive bacterium detected on the surface of smear-ripened cheese and other fermented foods.

## GENOME ANNOUNCEMENT

Staphylococci are Gram-positive catalase-positive bacteria with halotolerance that enables their growth in salted fermented food. Strains belonging to the *Staphylococcus* genus are commonly identified in soft cheese varieties made from cow, ewe, or goat milk, and together with *Brevibacterium*, *Corynebacterium*, and *Microbacterium*, *Staphylococcus* is considered the most important genus making up the microbiota of the cheese surface (1). *S. saprophyticus* is a species frequently detected on the surface of smear-ripened cheese and other fermented foods (2–4). Here, we present the draft genome sequence of *S. saprophyticus* DPC5671, which will allow a full safety assessment and further analysis on its role in cheese ripening.

The draft genome of *S. saprophyticus* DPC5671 was sequenced using paired-end 454 pyrosequencing to a coverage of 23×. Sequencing took place at the Teagasc 454 sequencing facility on a genome sequencer FLX platform (Roche Diagnostics, West Sussex, United Kingdom), according to the manufacturer’s protocols. This was followed by initial assembly into 24 contigs using the Newbler program (Roche Life Science). The software Prodigal (5) was used to predict open reading frames within the draft genome, and the RAST annotation server (6) was used to determine complementary gene calling and automated annotation. The draft genome was manually analyzed using the ARTEMIS genome browser (7), and comparative analysis with the genome of *S. saprophyticus* ATCC 15305 (8) was performed using the Artemis Comparison Tool (ACT) (9). The PHAST (PHAge Search Tool) Web server (10) was used to determine the presence of putative phage within the genome. The Comprehensive Antibiotic Resistance Database (CARD) software (11) was used to determine the presence of genes potentially involved in antibiotic resistance, and the presence of known staphylococcal virulence factors was analyzed using the BlastP Web server (12).

The draft genome of *S. saprophyticus* DPC5671 includes 2,676,318 bp, with an average G+C content of 33.1%. It consists of a single circular chromosome and does not appear to harbor any plasmids. Within the draft genome, there are 2,647 coding regions predicted, in addition to four rRNA and 59 tRNA genes. The genome sequence also includes two putative novel phages of ~43.6 kb and ~42.9 kb within the chromosome. Overall, the genome of *S. saprophyticus* DPC5671 shows high similarity to *S. saprophyticus* ATCC 15305 in genome size, G+C content, and gene synteny (8).

*S. saprophyticus* DPC5671 is coagulase negative, nonhemolytic, and does not appear to produce any toxins associated with *Staphylococcus aureus*. Genomic analysis revealed no obvious transferable antibiotic resistance loci by the methods used in this study. Previous studies showed an involvement of *S. saprophyticus* in urinary tract infections, showing a specific adhesin, *uafA*, to be associated with adherence to the eukaryotic cell in the urinary tract (8). The *S. saprophyticus* DPC5671 genome was found to have a predicted coding sequence (CDS) for adhesion, with similarities examined with the BlastP Web server to *uafA* in *S. saprophyticus* ATCC 15305 with 39% of query cover and 97% of protein identity.

The availability of the genome sequence of DPC5671 will allow its role in flavor development in cheese ripening to be studied.

### Accession number(s).

This whole-genome shotgun project has been deposited at DDBJ/ENA/GenBank under the accession number MUXI00000000. The version described in this paper is version MUXI01000000.
